# *Gliophorusglutinosus* sp. nov. (Hygrophoraceae, Agaricales) from Eastern Himalayan region of India

**DOI:** 10.3897/mycokeys.44.28554

**Published:** 2018-12-21

**Authors:** Dyutiparna Chakraborty, Kanad Das, Alfredo Vizzini

**Affiliations:** 1 Botanical Survey of India, Cryptogamic Unit, P.O. Botanic Garden, Howrah 711103, India; 2 Department of Life Sciences and Systems Biology, Univ. of Torino, Torino, Italy

**Keywords:** Agaricomycetes, Basidiomycota, *
Gliophorus
*, Hygrophoraceae, macrofungi, phylogeny, Sikkim, taxonomy

## Abstract

An interesting species of the genus Gliophorus (sect.Glutinosae), collected from Sikkim Himalaya in India, is described here as *G.glutinosus***sp. nov.** after thorough morphological examination and phylogenetic analysis. The species is mainly characterised by its strongly glutinous basidiomata throughout, particularly on the twisted stipe, decurrent lamellae with glutinous edge, gelatinised cheilocystidia, presence of pleuropseudocystidia and absence of clamps in hyphae of the pileipellis. This communication includes detailed morphological description, illustrations, comparison with the allied taxa, nrITS based phylogeny of this novel taxon and a key to the species under Gliophorussect.Glutinosae.

## Introduction

*Gliophorus* Herink (1958: 80) is a member of tribe Humidicuteae, subfamily Hygrocyboideae of Hygrophoraceae (Agaricomycetes, Basidiomycota) and featured by its glutinous basidiomata, variously coloured but never bright red; sinuate or decurrent lamellae, which are sometimes gelatinised at edge; basidiospores smooth, hyaline, thin-walled, inamyloid, ovoid to ellipsoid; presence or absence of ixocheilocystidia; basidia mostly 4-spored, presence of basal clamp in basidia and basidioles; irregular hymenial trama; pileipellis an ixotrichoderm (Lodge et al. 2014, Singh et al. 2017). This small genus (only 13 members were recorded under this genus in Index Fungorum, http://www.indexfungorum.org/names/Names.asp) encompasses 3 sections: sect.Gliophorus, sect.Glutinosae (Kühner) Lodge & Padamsee in Lodge et al. (2014: 6) and sect.UnguinosaeHerink (1959: 81) (Lodge et al. 2014). Gliophorussect.Glutinosae, typified by *Gliophoruslaetus* (Persoon 1800: 48) Herink (1959: 84), is further characterised by plano-convex pileus that is often indented in centre; green, olive, blue, violet, pink, salmon, yellow, buff, orange or orangish-brown coloured pileus; decurrent lamellae with gelatinised edge; cheilocystidia usually present and embedded in gelatinous matrix (ixocheilocystidia); basidiospores mostly binucleate.

During a macrofungal survey and collection tour to different forested areas of South Sikkim, two of us (DC & KD) came across a very interesting and tiny member of Gliophorussect.Glutinosae. After detailed macro- and micromorphological characterisation, coupled with the phylogenetic studies based on the sequence data of nuclear ribosomal internal transcribed spacer (nrITS) region of that species, it was shown to be distinct from any other known species in *Gliophorus* and is proposed here as *G.glutinosus* sp. nov. Detailed morphological description, supporting illustrations and phylogenetic inference is presented here for this novel species.

## Material and methods

### Morphological study

Macromorphological characters were recorded in the forest and in base-camp from two collections of 13 fresh and dissected young to mature basidiomata. Images of the fresh basidiomata were captured with a Canon Power Shot SX 50 HS. Colour codes and terms are mostly after Methuen Handbook of Colour (Kornerup and Wanscher 1978). Micromorphological characters were observed with a compound microscope (Nikon Eclipse Ni-U). Sections from dried specimens were mounted in a mixture of 5% potassium hydroxide (KOH), 1% Phloxine and 1% Congo red or in distilled water. Micromorphological drawings were prepared with a drawing tube (attached to the Nikon Eclipse Ni) at 1000× magnification. The basidium length excludes sterigmata. Basidiospore measurements were recorded in profile view from 30 basidiospores. Spore measurements and length/width ratios (Q) are recorded here as: minimum–*mean*–maximum. Herbarium codes follow Thiers 2018 (continuously updated).

### DNA extraction, PCR amplification and sequencing

Genomic DNA was extracted from dried herbarium specimens (100 mg) using the XcelGen Fungal gDNA Mini Kit (Xcelris Genomics, Ahmedabad, India). The nuclear ribosomal ITS region was amplified using the primers ITS1F and ITS4 (White et al. 1990). Amplification (with PCR) was performed in a 50 μl reaction mix comprising 2 μl template DNA (10–20 ng), 0.5 U Taq DNA polymerase (Sigma-Aldrich, India), 5 μl 10X Taq DNA polymerase buffer, 1 μl 200 μM of each dNTP (Sigma-Aldrich, India), 1 μl 10 pmol primer and the remaining volume made up by H_2_O (Sterile Ultra Pure Water, Sigma-Aldrich). This amplification was done using an Eppendorf Mastercycler (Eppendorf, Hamburg, Germany) with the following parameters: 5 min step at 95 °C, followed by 30 cycles of 1 min at 95 °C, 30 s at 55 °C and 1 min at 72 °C and a final 7 min extension step at 72 °C. Products from PCR were then purified with QIAquick PCR Purification Kit (QIAGEN, Germany) and sequenced using the BigDye Terminator v3.1 Cycle Sequencing Kit (Applied Biosystems, USA). The sequencing products were run on 3730×l DNA Analyzer (Applied Biosystems, USA). The raw DNA files were edited and combined using FinchTV and ChromasLite v. 2.01. The sequences generated from two collections (DC 17–28 and DC 17–38) were deposited in GenBank (MH392195 and MH392196).

### Phylogenetic analysis

**Figure 1.**
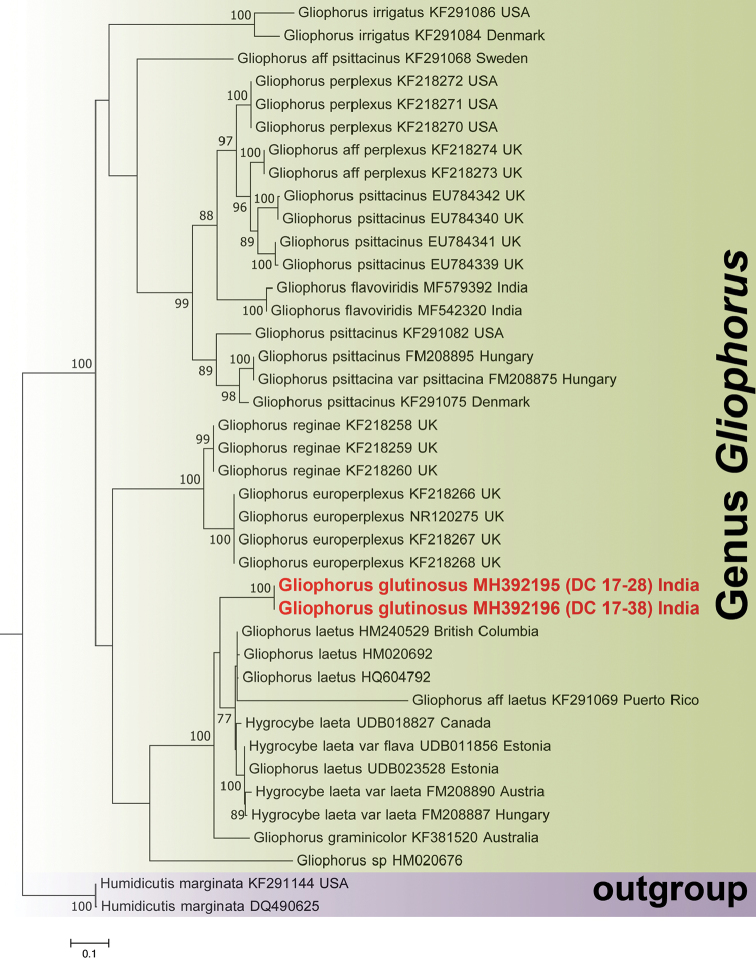
Phylogram resulting from Maximum likelihood analyses of nrITS region. Nodes were annotated with MLB values. MLB values > 70% are shown. Sequences derived from the novel species *Gliophorusglutinosus* (MH392195 and MH392196) are shown in red and bold.

**Figure 2.**
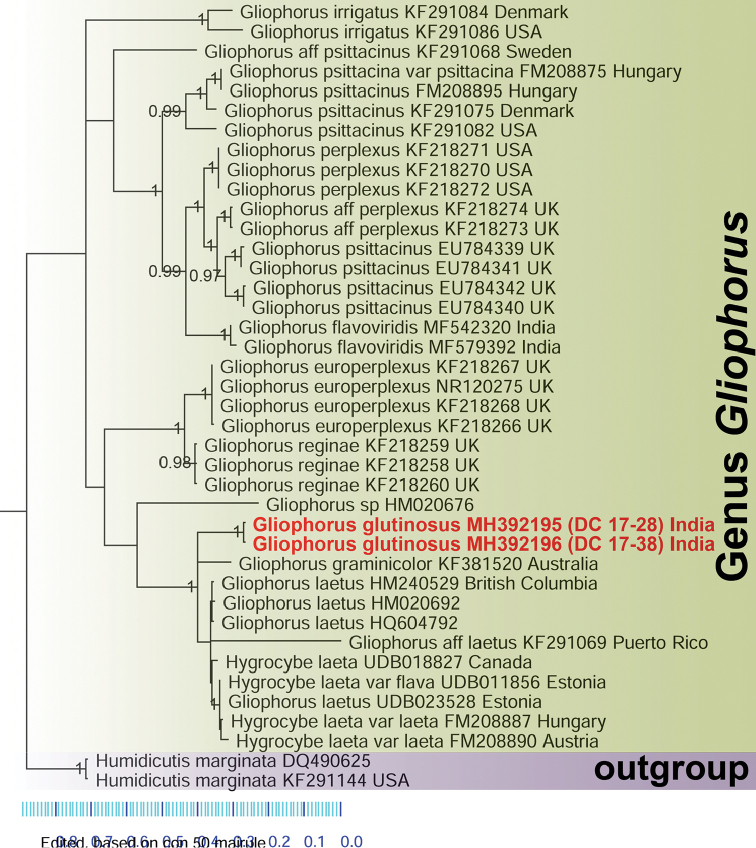
Phylogram resulting from Bayesian phylogeny of nrITS region. Nodes were annotated with BPP values. BPP values > 0.95 are shown. Sequences derived from the novel species *Gliophorusglutinosus* (MH392195 and MH392196) are shown in red and bold.

Phylogenetic analyses were based on internal transcribed spacer (ITS) nuclear ribosomal DNA sequences data to establish the phylogenetic placement of the new species. Datasets including reference sequences and outgroup were prepared following relevant literature (Ainsworth et al. 2013, Lodge et al. 2014, Singh et al. 2017), BLAST searches (Altschul et al. 1997) and data retrieved from public databases such as GenBank (Clark et al. 2016) and UNITE (Kõljalg et al. 2013). Multiple sequence alignment was performed using MAFFT v.7 (Katoh and Standley 2013). The aligned loci were loaded in PAUP* 4.0b 10 (Swofford 2001) and the best-fit substitution model of nucleotide evolution (GTR+I+G) was carried out in MrModeltest 3.7 (Posada and Crandall 1998). Bayesian inference was computed in MrBayes v.3.2.2 (Ronquist et al. 2012). Bayesian posterior probabilities (BPP) were calculated in two simultaneous runs with the Markov chain Monte Carlo (MCMC) algorithm (Larget and Simon 1999). Markov chains were run for 1000000 generations, saving a tree every 100^th^ generation. Default settings in MrBayes were used for the incremental heating scheme for the chains (3 heated and 1 cold chain), unconstrained branch length [unconstrained: exponential (10.0)] and uninformative topology (uniform) priors. The analysis was allowed to terminate when the average standard deviation of split frequencies was below 0.01. The first 25% of trees was discarded as burn-in (Hall 2004). Simultaneously, with the same dataset, a full search for the best-scoring Maximum likelihood tree was conducted in RAxML (Stamatakis 2006) using the standard search algorithm (ITS1-5.8S-ITS2 data partitioned, 1000 bootstrap replications). The significant threshold was set above 0.95 for Bayesian posterior probability (BPP) and above 70% for Maximum likelihood bootstrap support (MLB). Phylograms (Figs 1, 2), inferred from Maximum likelihood method and Bayesian phylogeny, are presented showing MLB and BPP values, respectively, for the eligible branches.

## Results

### Phylogeny

The nrITS-sequence dataset consists of 40 sequences. In the Maximum likelihood analysis (Fig. 1), the two Indian collections of the proposed novel species, *Gliophorusglutinosus* (MH392195-DC 17–28 and MH392196-DC 17–38) clustered together (MLB = 100%) as a distinct species and appeared sister to the clade (MLB = 77%) bearing *G.laetus* from Europe, North America, Puerto Rico and Austria (HM240529, HM020692, HQ604792, KF291069, UDB018827, UDB011856, UDB023528, FM208887, FM208890). In turn, *G.laetus* and *G.glutinosus* clustered together as sister (MLB = 100%) to an Australian collection of *G.graminicolor* E. Horak (1973: 176) (KF381520). Similarly, in our Bayesian phylogeny (Fig. 2), the two Indian collections of *Gliophorusglutinosus* clustered together (BPP = 1.00) and appeared sister to the clade bearing *G.laetus* from Europe, North America, Puerto Rico and Austria (HM240529, HM020692, HQ604792, KF291069, UDB018827, UDB011856, UDB023528, FM208887, FM208890). The Australian collection of *G.graminicolor* (KF381520) also appeared as nested (without ancestral information) between the *G.laetus* cluster and *G.glutinosus*.

### Taxonomy

#### Gliophorus glutinosus

Taxon classificationFungiAgaricalesHygrophoraceae

K. Das, D. Chakr. & Vizzini sp. nov.

825657

Figs 3 , 4

##### Diagnosis.

Distinguished from all the allied taxa by its nrITS sequence and possessing a combination of features like typically twisted stipe submerged under thick gluten, sticky pileus, presence of gluten at lamellar edge, decurrent lamellae, indistinct odour, ixocheilocystidia and presence of pleuropseudocystidia and absence of clamps in pileipellis hyphae.

##### Type.

INDIA. Sikkim: South District, Thangse, 1962 m alt., 27°18.496'N, 88°21.519'E, 23 August 2017, *D. Chakraborty* & *K. Das*, *DC 17–28* (Holotype CAL!).

##### Etymology.

The epithet “*glutinosus*” refers to the highly glutinous stipe surface.

*Pileus* 5–20 mm diam., convex with a shallow central depression at disc when young, becoming plano-convex at maturity; surface highly glutinous, sticky, sulcate-striate, greyish-orange (6C−B5), brownish-orange (5C5), becoming pale orange to orange white (7C7, 6A3−2) with maturity, sometimes whitish to pastel yellow (2A4) at centre; margin crenate; context ≤ 2 mm thick, concolorous with pileus surface. *Lamellae* subdecurrent to decurrent, moderately close to subdistant (11 per 10 mm at pileus margin), viscid, pale orange to orange white (5A3−2); lamellulae in 3 series; edges glutinous, concolorous with face of lamellae, viscid. *Stipe* 10−60 × 2−5 mm, central, hollow, cylindrical, often gradually broaden towards base, twisted, longitudinally furrowed, submerged under thick sticky gluten (1 mm); surface upper half pale orange (5A3) and pale yellow to light yellow (4A3−4) towards base. *Taste* and *odour* indistinct. *Spore print* not obtained.

*Basidiospores* 6−*7*−8 × 3−*4.1*−5 μm (n = 30, Q = 1.5−*1.72*−2.16), elongate-ellipsoid to nearly cylindric, smooth, thin-walled, hyaline, inamyloid, uni- to multiguttulate. *Basidia* 30–38 × 5–7 μm, clavate, thin-walled, with a basal clamp-connection, 2- to 4-spored; sterigmata up to 10 μm long. *Lamellar edge* sterile. *Cheilocystidia* 35–62 × 2–5 μm, slender, occasionally septate, mostly clustered together, gelatinised (embedded in gelatinous matrix). *Pleuropseudocystidia* 31–40 × 5–7 μm, rare, subclavate to appendiculate or fusoid. *Subhymenium* 16–23 µm thick, not gelatinised. *Hymenophoral trama* subregular, consisting of clamped hyphae (3–10 μm diam.), terminal and subterminal cells 17–48 µm long, terminal cells often inflated. *Pileipellis* an ixocutis (when mounted in water or cotton blue), 25−60 μm thick, submerged under thick gluten (seen when mounted with cotton blue), composed of suberect, thin-walled, septate and frequently branched hyphae (observed when mounted in 5% KOH making it free from gluten); terminal elements 15–40 × 2–5 μm, with rounded apex, clamps absent. *Stipitipellis* an ixocutis (when mounted in water or cotton blue) to an ixotrichoderm (when revived in KOH), mostly similar to that of pileipellis.

##### Habitat/ Distribution.

Growing in groups or gregariously on soil amongst leaf-litter of angiospermous plants.

##### Additional specimen examined.

INDIA. Sikkim: South District, Thangse, 1962 m alt., 27°18.496'N, 88°21.519'E, 23 August 2017, *D. Chakraborty* & *K. Das*, DC 17–38 (CAL).

**Figure 3.**
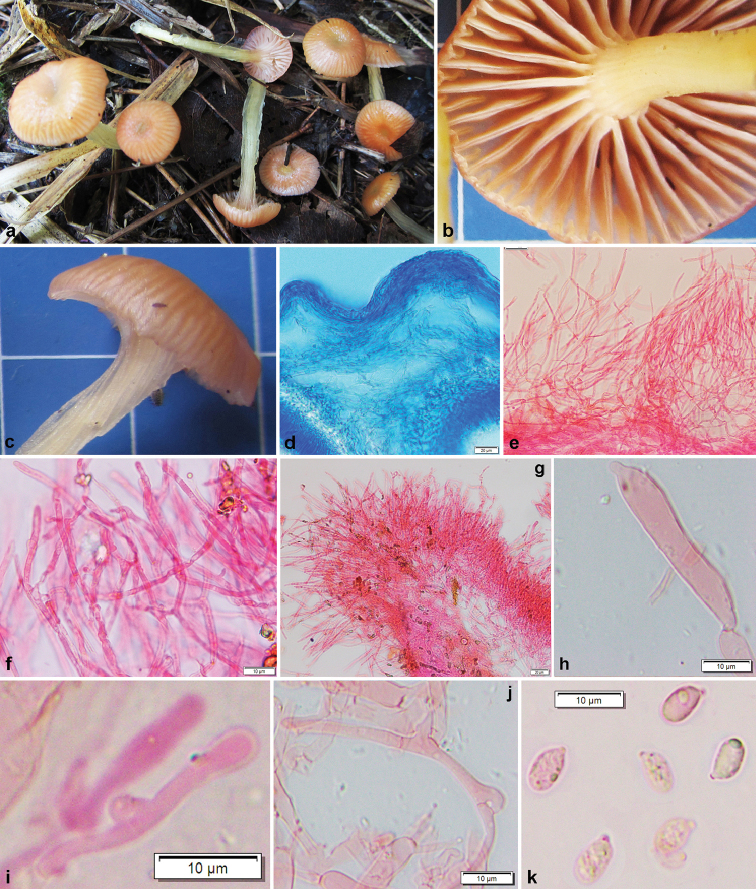
*Gliophorusglutinosus* (from DC 17–28, holotype). **a–c** Fresh basidiomata in field or in base-camp **d** Transverse section through pileipellis showing ixocutis pattern (under cotton blue) **e, f** Hyphal elements in pileipellis (after removal of gluten) **g** Cheilocystidia (after removal of gluten at lamellae edge) **h** Pleuropseudocystidium **i** Basidioles **j** Hyphae in hymenophoral trama**k** Basidiospores. Scale bars: 20 µm (**d, e, g**); 10 µm (**f, h, i, j, k**).

**Figure 4.**
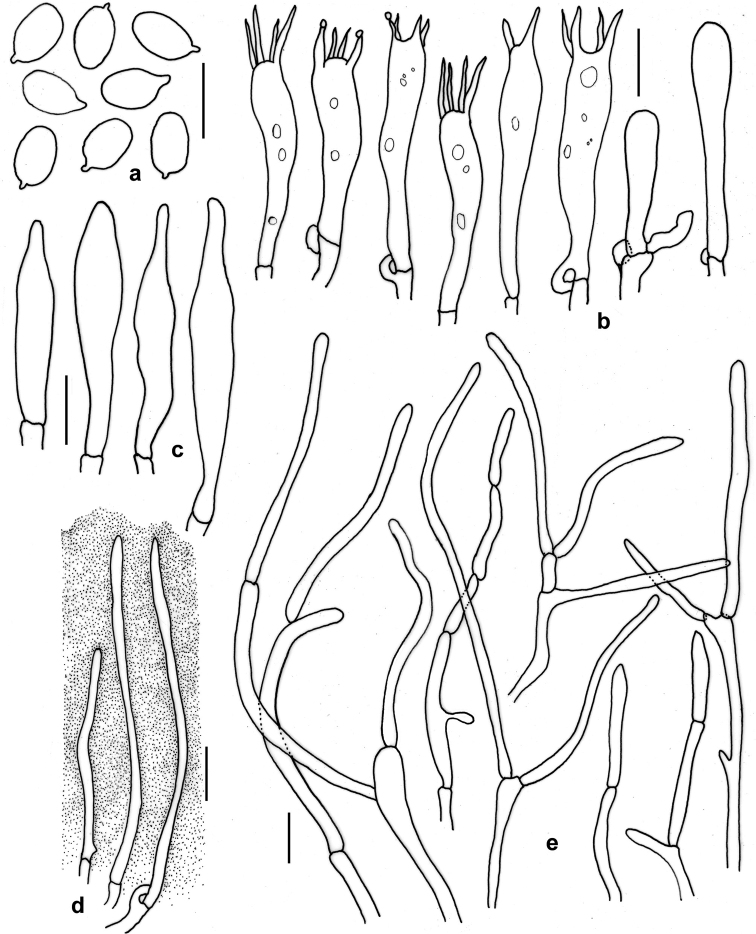
*Gliophorusglutinosus* (from DC 17–28, holotype) **a** Basidiospores **b** Basidia and basidioles **c**Pleuropseudocystidia**d** Ixocheilocystidia **e** Hyphal elements in pileipellis (without gluten and in 5% KOH). Scale bars: 10 µm (**a–e**).

## Discussion

The combination of features, such as significantly sticky small basidiomata, distinctively twisted stipe which is completely submerged within a thick (1 mm) transparent layer of gluten, decurrent lamellae with glutinous (sticky) edges, presence of pleuropseudocystidia (sterile elements arising deep in the hymenophoral trama and protruding into the hymenium) and absence of clamps in hyphae of pileipellis, separate *G.glutinosus* from all the known species of *Gliophorus*. Features, such as decurrent lamellae with sticky edges, planoconvex to slightly depressed pileus and presence of ixocheilocystidia, placed the Indian collection under Gliophorussect.Glutinosae. In fact, in the phylogenetic analysis (Figs 1–2), the new species forms a strongly supported clade together with *G.laetus*, type species of the sect.Glutinosae and with *G.graminicolor*. To our best knowledge, this is the first report of the presence of pleuropseudocystidia in a *Gliophorus* species or, in general, in *Hygrocybe* s.l. (Singer 1986, Boertmann 2010, Lodge et al. 2014). So far, only cheilopseudocystidia have been described as present, albeit rarely, in *Hygrocybe* s.l. (Boertmann 2010, Lodge et al. 2014).

Morphologically, *G.glutinosus* is similar to *G.laetus* [≡*Hygrocybelaeta* (Pers.) P. Kumm. (1871: 112); ≡*Hygrophoruslaetus* (Pers.) Fries 1838: 329] but the latter differs by having significantly larger basidiomata (pileus 10–50 mm diam., stipe 30–120 mm long), stipe which is never twisted and less glutinous and showing greyish-lilac tinges at apex; a strongly gelatinised and up to 140 μm thick subhymenium, presence of cuticular clamped hyphae and having an unpleasant odour, described as like burned rubber, burned hair, fish or animal cages (Hesler and Smith 1963, Arnolds 1974, 1990, Boertmann 2010, Bessette et al. 2012). *Gliophorusgraminicolor* E. Horak [≡*Hygrocybegraminicolor* (E. Horak) T.W. May & A.E. Wood 1995: 148] from Australia (Tasmania included) and New Zealand is though genetically close to this novel Indian species and can be separated by possessing brown to greenish-brown or grass green coloured pileus and stipe, less viscid stipe, odour and taste unpleasant, like burnt hair, presence of clamps in pileus hyphae (Horak 1973, 1990, Young and Wood 1997 as *Hygrocybebatesii* A.M. Young (in Young and Wood 1997: 956), Young 1999, 2005, Young and Mills 2002). *Hygrocybenoelokelani* Desjardin & Hemmes (1997: 621), from Hawaii, shows a deep pink, pastel red or pale red pileus, a non-twisted, less viscid stipe, ovoid to broadly ellipsoid, spores (up to 6 μm wide) and presence of large clamp-connections on pileipellis hyphae (Desjardin and Hemmes 1997). *Hygrocybecorallina* Leelav., Manim. & Arnolds (2006: 125), from Kerala, India, has pale red to coral-red basidiomata with bright red lamellae, larger spores [7–10(–11) × 4.5–6.5 μm], clamps observed in all parts of basidioma and the hymenophoral trama regular, made up of medium-sized to long, thin-walled elements, 100–500 × 3–20 μm (Leelavathy et al. 2006).

## Key to the species in Gliophorussect.Glutinosae worldwide (* indicated species included in the section are based on morphology alone)

**Table:** 

1	Pileipellis as ixocutis	**2**
–	Pileipellis as ixotrichoderm	**6**
2	Pileus whitish to pale argillaceous; lamellae adnate	*G.pallidus* E. Horak*
–	Pileus dark and/or bright coloured; lamellae decurrent	**3**
3	Pileus reddish-brown or lilac pink, liver brown; cheilocystidia absent	*G.versicolor* E. Horak*
–	Pileus greenish-blue, green or orange-brown; cheilocystidia present	**4**
4	Pileus orange-brown, strongly glutinous; stipe twisted, embedded in a thick layer of gluten; basidia 2- to 4-spored	***G.glutinosus* (DC 17–28)**
–	Pileus green to greenish-blue, surface moderately glutinous; stipe equal; basidia 4-spored	**5**
5	Pileus green; lamellae whitish with greenish tinge; odour burnt-hair like, unpleasant	*** G. graminicolor ***
–	Pileus greenish-blue, turning bluish-lilac with age; lamellae lilac blue or pale greenish-blue; odour none	*G.lilacipes* E. Horak*
6	Pileus pinkish-orange, orange brown or pastel red to pale red; lamellae sub-decurrent to decurrent	**7**
–	Pileus green or yellow; lamellae broadly adnate	**9**
7	Clamp-connections in pileipellis frequent	*** G. laetus ***
–	Clamp-connections in pileipellis very rare	**8**
8	Pileus orange brown, paler with age; lamellae decurrent, pale yellow; stipe tapering down or broader at middle	*H.viscidibrunnea* Bougher & A.M. Young*
–	Pileus pink or pastel red or pale red; lamellae subdecurrent, pale pinkish-white; stipe cylindrical	H.noelokelani*
9	Pileus greenish; lamellae greenish	*G.pseudograminicolor* A.M. Young*
–	Pileus yolk yellow to lemon yellow; lamellae yellow	*G.chromolimoneus* (G. Stev.) E. Horak*

## Supplementary Material

XML Treatment for Gliophorus glutinosus
